# Apple Pomace Fermented with Non-*Saccharomyces* Yeast as a Factor Modulating Gut Microbiota

**DOI:** 10.3390/ijms27072960

**Published:** 2026-03-24

**Authors:** Wiktoria Liszkowska-Walisiak, Ilona Motyl, Barbara Płacheta-Kwiatkowska, Małgorzata Wlaźlak, Tomasz Ruman, Joanna Nizioł, Agnieszka Wilkowska, Agnieszka Maher, Joanna Berłowska

**Affiliations:** 1 Department of Environmental Biotechnology, Faculty of Biotechnology and Food Sciences, Lodz University of Technology, 171/173 Wólczańska Street, 90-530 Łódź, Poland; 2 Institute of Molecular and Industrial Biotechnology, Faculty of Biotechnology and Food Sciences, Lodz University of Technology, 2/22 Stefanowskiego Street, 90-537 Łódź, Poland; 3 Department of Inorganic and Analytical Chemistry, Faculty of Chemistry, Rzeszów University of Technology, 6 Powstańców Warszawy Ave, 35-959 Rzeszów, Poland; 4 Department of Polymers and Biopolymers, Faculty of Chemistry, Rzeszów University of Technology, 6 Powstańców Warszawy Ave, 35-959 Rzeszów, Poland; 5 Institute of Fermentation Technology and Microbiology, Faculty of Biotechnology and Food Sciences, Lodz University of Technology, 171/173 Wólczańska Street, 90-530 Łódź, Poland; agnieszka.wilkowska@p.lodz.pl

**Keywords:** apple pomace valorisation, bioactive component, low-temperature fermentation, gut microbiota, metagenomics, metabolomics

## Abstract

The valorisation of agro-industrial by-products through fermentation offers an opportunity to develop functional ingredients with targeted effects on gut microbiota. This study evaluates the impact of apple pomace fermented at a low temperature (15 °C) by cold-adapted yeast on the structure and metabolic activity of human gut microbiota, simulated using the Simulator of the Human Intestinal Microbial Ecosystem (SHIME^®^). The fermented apple pomace preparation was characterised by high stability under gastrointestinal conditions, supporting its potential applicability as a functional food ingredient. Supplementation with fermented apple pomace induced distinct changes in the composition and activity of gut microbiota compared to the non-fermented substrate, including increased abundance of the genera *Akkermansia*, *Coriobacteriaceae*, and *Parabacteroides*, and reduced abundance of *Bifidobacterium*, *Klebsiella*, *Serratia*, and *Raoultella*. The fermented preparation was associated with reduced accumulation of metabolites typically linked to proteolytic fermentation and a more stable metabolic profile throughout the supplementation and washout phases. Short-chain fatty acid analysis indicated that fermentation influenced both the quantity and proportional balance of microbial fermentation products, promoting profiles closer to physiological reference ranges. Overall, fermentation of apple pomace at 15 °C enhanced its functional properties and modulated gut microbiota metabolism in a manner consistent with improved ecosystem stability. These findings highlight the potential of fermented fruit by-products as sustainable ingredients for dietary strategies aiming to support gut microbial functionality.

## 1. Introduction

The gut microbiota plays a fundamental role in maintaining human health, influencing metabolism, the immune system, and even neurological processes. The microbiota consists of a complex community of microorganisms, including bacteria, archaea, fungi, and viruses, which interact with the host and with each other to maintain a balanced ecosystem [1,2]. Imbalance in this microbial community, known as dysbiosis, has been linked to various chronic diseases, including inflammatory bowel disease, obesity, and diabetes [3,4].

Dietary components are the most significant modulators of the gut microbiota. The composition and functionality of the gut microbiota can be significantly altered by the consumption of particular dietary components, including fermented foods and fibre-rich substrates [5]. Fermented products and foods rich in dietary fibre are known to enhance microbial diversity and promote the growth of beneficial microorganisms [6,7]. These effects are largely attributed to bioactive components, such as prebiotics (which serve as substrates for gut microbes) and probiotics (which directly introduce beneficial microorganisms into the gut, for example by stimulating the production of short-chain fatty acids) [8,9]. Prebiotics are nondigestible fibre compounds that pass undigested through the upper part of the gastrointestinal tract. They promote the growth and activity of beneficial microorganisms. Probiotics support the colonisation of the intestinal region and stimulate the growth of other probiotic bacteria in the gut. This is beneficial to the host, while being harmful to pathogenic bacteria [10,11]. Probiotics include Gram-positive and Gram-negative bacteria, bacteriophages, microalgae, and yeasts [10,12]. Yeasts possess anti-inflammatory effects, enhance intestinal barrier function, and inhibit pathogen colonisation. Furthermore, they contribute to fermentation processes, enhancing the bioavailability of nutrients and producing metabolites with prebiotic and antimicrobial properties [13].

The most well-known probiotic yeast, *Saccharomyces cerevisiae* var. *boulardii*, has been shown to inhibit pathogens, modulate immune responses, and help maintain intestinal barrier integrity [14]. Currently, *S. boulardii* is the only yeast strain widely used and clinically validated in commercial probiotic preparations. Other microorganisms commercialised as probiotics include *Saccharomyces cerevisiae*, *Kluyveromyces fragilis*, and *Kluyveromyces marxianus* [15]. The limited number of microorganisms currently used in commercial probiotic preparations highlights the potential of naturally derived formulations produced from biomass fermented by environmental yeast strains. These formulations could deliver viable microorganisms within a bioactive fermented matrix enriched with organic acids, partially degraded polysaccharides, and volatile aromatic compounds that synergistically enhance microbial stability and gut health efficacy. Yeast-fermented food matrices improve probiotic viability during storage and gastrointestinal transit compared with isolated cells. Additionally, matrix-derived metabolites (e.g., phenolic compounds and short-chain fatty acids, SCFAs) exhibit anti-inflammatory and prebiotic effects in vivo [14].

The most important factors for selecting an appropriate substrate for food development are cost and availability, making agro-industrial residues attractive candidates [16]. Apple pomace, a by-product of apple juice production, is abundant in dietary fibres and polyphenols, making it a potential functional ingredient for gut health [17]. Its incorporation into the diet has been associated with antioxidant, anti-inflammatory, and prebiotic effects, supporting the growth of beneficial gut microbiota and enhancing SCFA production [18]. The management of production waste has become a priority for many industrial sectors. In the European Union, over 2.2 billion tons of waste are generated annually [19]. As a result, there is an ongoing search for eco-friendly solutions that allow for the utilisation of production residues. Aligned with the principles of the circular economy, efforts are being made to keep materials and resources in circulation for as long as possible. One approach is fermentation, which is a natural method for transforming complex plant-based substrates into more accessible components.

This study evaluates the impact of bioactive components derived from apple pomace fermented at low temperatures on human gut microbiota. Low-temperature fermentation ensures that the obtained bioferment has greater stability and shows increased retention of thermolabile compounds [20]. Cold-adapted yeast strains, originally isolated from a plant-based environment, were tested for their ability to survive under intestinal passage conditions. The most resistant yeast was then used to ferment apple pomace at 15 °C. The impact of the fermented apple pomace on the structure and metabolic activity of human gut microbiota was then assessed in a Simulator of the Human Intestinal Microbial Ecosystem (SHIME^®^).

## 2. Results and Discussion

Prior to the digestion tests, the fermentation dynamics of selected yeast strains at low temperatures (15 °C) were characterised in apple pomace. Their growth peak was reached after 5 days (~10^8^ CFU/mL), with efficient consumption of fermentable sugars and production of organic acids. The biosynthesis of volatile organic compounds enhanced the sensory profile of the fermented substrate [21].

### 2.1. Viability of Yeast Under Gastrointestinal Tract Conditions

**Figure 1:**
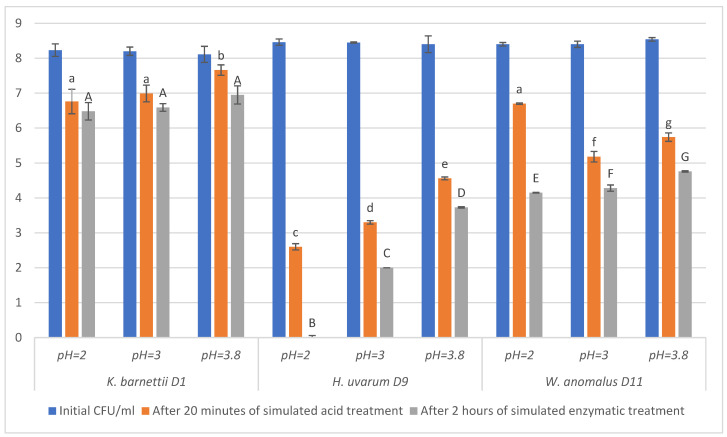
INFOGEST results for the tested yeast strains. Blue bars indicate the initial CFU/mL of microorganisms in the tested samples; orange bars show the CFU/mL after 20 min of acid treatment; grey bars indicate the CFU/mL after enzymatic treatment. Lowercase letters indicate significant differences in the values of the measured parameters for samples after simulated acid treatment and capital letters indicate significant differences in the values of the measured parameters for samples after simulated enzymatic treatment (*p* ≤ 0.05).

Figure 1 presents the viability of the tested yeast strains under different conditions of pH and enzymatic treatment. As the pH value after fermentation was around 3.8, this was set as the highest pH value in the experiment.

The viability rate was the highest for all three yeast strains at pH = 3.8. *Kazachstania barnettii* D1 and *Wickerhamomyces anomalus* D11 showed superior tolerance to highly acidic environments. After 20 min of exposure at pH 2–3, viable counts decreased by approximately two log units. In contrast, *Hanseniaspora uvarum* D9 demonstrated extreme sensitivity to acidic environments, particularly at pH = 2 and pH = 3, at which its viability dropped drastically. At pH = 2, the number of cells decreased by six logarithmic units after 20 min of acid exposure. At pH = 3, the number of cells decreased by five logarithmic units after acid treatment, indicating poor tolerance to gastric-like acidity.

After gastric exposure, yeasts encounter bile salts, pancreatic enzymes, and alkaline pH in the small intestine, which together constitute strong physiological stressors. Bile salts disrupt membranes, while digestive enzymes degrade cellular structures, allowing the survival only of strains equipped with effective stress-response mechanisms [22]. Although *S. cerevisiae* yeast survives in acidic environments, it struggles in highly alkaline conditions. On the other hand, species such as *Yarrowia lipolytica* survive at a pH up to 10–11 [23,24,25,26]. The two-hour enzymatic treatment applied in this study, designed to simulate intestinal conditions, revealed differences in strain viability. Across all tested pH values, *K. barnettii* D1 consistently maintained the highest viability, followed by *W. anomalus* D11. In contrast, *H. uvarum* D9 showed pronounced inhibition. It can be inferred that *K. barnettii* D1 and *W. anomalus* D11 possess strong cell wall integrity and membrane remodelling systems, enabling them to survive acidic and enzymatic stress, while *H. uvarum* D9 lacks such adaptations [22,27,28,29].

The ability of yeasts to survive and maintain biological activity under gastrointestinal conditions is a crucial factor determining their potential probiotic features and uses as functional ingredients in food products [30]. These conditions are characterised primarily by the abundance of gastric acids, bile salts, and digestive enzymes. A highly selective environment is created, in which only resistant microorganisms can survive and maintain biological activity. Therefore, in order to evaluate the probiotic potential of yeast strains, it is crucial to evaluate them under simulated gastrointestinal conditions [31]. Fermentation-derived yeasts, including those isolated from plant-based environments, may exhibit strain-specific adaptations that influence their gastrointestinal resistance. For instance, cold-adapted yeasts possess unique stress-response mechanisms that can improve viability under demanding pH conditions in the digestive tract [20]. A key element of this adaptation is the modification of the cell wall, which is primarily achieved through the activation of the cell wall integrity signalling pathway [32]. In response to stress induced by strong hydrochloric acid present in gastric juice, carbohydrate polymers are synthesised and the cell wall is strengthened [23]. A thicker cell wall made of β-glucans and mannoproteins provides yeast with structural integrity, and consequently, resistance to acidic and enzymatic degradation. Additionally, a general stress response pathway involving protein kinase C is activated to coordinate overall metabolic adjustment to environmental conditions [33]. Another important survival mechanism is the regulation of membrane lipid composition. Changes in lipid profile, especially sterols, influence membrane fluidity and integrity, enhancing yeast acid tolerance. Changes in membrane lipids have been shown to significantly increase acid resistance at low pH up to 2.0 [34].

*K. barnettii* D1 displayed the highest viability across all tested pH levels and after enzymatic exposure, indicating strong resistance mechanisms to both acidic and intestinal stresses. *W. anomalus* D11 also showed moderate viability, suggesting potential as a probiotic strain [35]. In contrast, *H. uvarum* D9 did not exhibit adaptation mechanisms under the analysed conditions, with low viability rates and sensitivity to gastrointestinal conditions. Overall, *K. barnettii* D1 exhibited the highest resistance to gastrointestinal conditions, indicating its potential as a probiotic in functional food applications. This strain was therefore chosen for further experiments.

### 2.2. Metagenomic Profiling of Gut Microbiota During 4-Week Supplementation with Fermented and Unfermented Apple Pomace

The human gut microbiota is a complex anaerobic ecosystem. Its composition is strongly influenced by dietary patterns. Dietary patterns are known to modulate the intestinal microflora, enhancing or supressing the production of key metabolites by microorganisms including SCFAs. Of particular interest is the diversity of microbiota in the large intestine. A healthy gut has a stable microbiological core, with the most common bacteria from *Bacteroidetes* and *Firmicutes* phyla [36].

**Figure 2:**
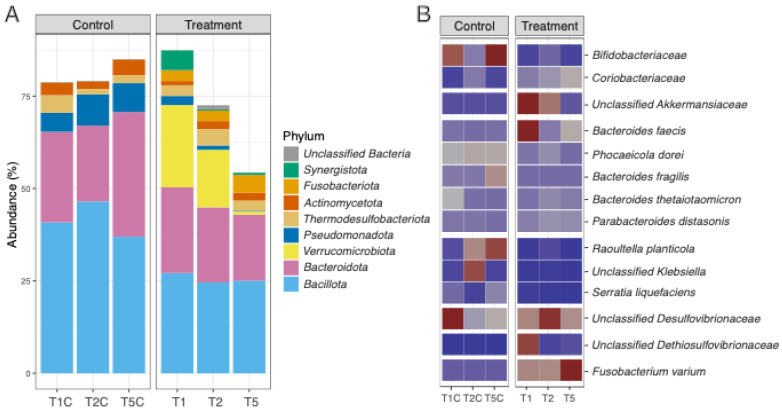
Analysis of gut microbiota composition: (**A**) relative abundance at phylum level for species-level representative genomes (SRGs); (**B**) heatmaps of relative abundance of selected families or species of the phyla. Colours indicate increases (red) and decreases (blue) with respect to mean normalised values in each phylum. Detailed values for abundance are displayed in the Supplementary Materials Figure S1. Abbreviations: T1C—control sample from the first week of the main phase of the experiment; T2C—control sample from the second week of the main phase of the experiment; T5C—control sample from the last day of the main phase of the experiment before the silencing phase; T1—the first week of the main phase of the experiment; T2—the second week of the main phase of the experiment; T5—the last day of the main phase of the experiment, before the silencing phase.

In this study, the taxonomic composition of the experimental group was compared to the control group, with special focus on temporal changes and responses to supplementation. Distinct differences were observed between the control (non-fermented pomace) and treated samples (fermented pomace) in terms of their impact on the structure of the microbiota. Analysis of the gut microbiota composition, shown in Figure 2A (as well as in Figure S1A–H in the Supplementary Materials), revealed major changes in the relative abundance of different phyla. Across all samples, *Bacillota* and *Bacteroidota* were the dominant phyla (43–71% of the community). Other phyla, including *Pseudomonadota*, *Actinomycetota*, *Verrucomicrobiota*, *Synergistota*, *Fusobacteriota*, and *Thermodesulfobacteriota*, each accounted for 37–11% of the community. A clear difference in microbiota structure was also visible in the control samples (supplemented with non-fermented apple pomace) compared to the research samples (supplemented with fermented apple pomace).

Supplementation with fermented pomace altered the relative abundance of major phyla. The modified *Bacillota*-to-*Bacteroidota* ratio compared to the control group indicates a reshaping of ecological niches due to substrate pre-fermentation. However, a gradual decrease in the abundance of *Verrucomicrobiota* was also observed in the treatment samples, occurring dynamically during the testing period. In comparison, the gut microbiota of the control samples remained more constant. These findings indicate that the fermentation process influences the prebiotic quality of apple pomace, promoting a gut microbial profile often associated with favourable metabolic outcomes. The reduced abundance of *Bacillota* in the treatment samples suggests that the fermentation activity of *K. barnettii* D1 alters the ecological niche available to *Bacillota*. Many representatives of this phylum, including butyrate-producing *Lachnospiraceae* and *Ruminococcaceae*, depend on complex carbohydrates and plant-derived polysaccharides as their primary energy sources. Fermentation of apple pomace by *K. barnettii* D1 can partially degrade complex plant polysaccharides and reduce the content of easily fermentable sugars, while introducing low-molecular-weight organic acids and phenolics, creating less favourable conditions for strictly anaerobic fibre-degrading microorganisms [37]. Similar reductions in *Bacillota* have been reported previously during polyphenol-rich or fermentation-modified diets and are attributed to both substrate limitation and the inhibitory effects of phenolic intermediates [38,39].

An increase in the abundance of *Coriobacteriaceae* was observed in the samples supplemented by fermented apple pomace (Figure 2B and Figure S1F in the Supplementary Materials). These microorganisms possess activity for the metabolism of polyphenols, aromatic compounds, and other fermentation-derived intermediates. They can also grow more efficiently when they have access to polyphenol-derived substrates and when cross-feeding networks among saccharolytic taxa weaken [40]. Their proliferation in the presence of fermented apple pomace therefore suggests an enhanced niche for microbial transformation of aromatic metabolites, consistent with observations from dietary studies involving flavonoids and other phytochemicals [41]. Interestingly, a decrease in *Bifidobacteriacae* following exposure to fermented relative to non-fermented pomace was observed. This may be related to the limited availability of oligosaccharides, which typically support bifidogenic growth (Figure 2B and Figure S1F in the Supplementary Materials). Moreover, increases in phenolic metabolites and fermentation-derived aromatic acids can inhibit the growth of some *Bifidobacterium* species, while promoting taxa with broader metabolic flexibility such as *Coriobacteriaceae* [42,43].

Three groups were particularly interesting: *Bacteroidota*, the genus *Akkermansia*, and *Pseudomonadota*. *Akkermansia* was detected exclusively in the models supplemented with fermented apple pomace (Figure 2B and Figure S1C in the Supplementary Materials), whereas *Bacteroides fragilis* predominated in the systems receiving the non-fermented preparation (Figure 2B and Figure S1B in the Supplementary Materials). This contrast reflects the distinct ecological and metabolic preferences of these taxa. Such conditions favoured microorganisms adapted to a nutrient-limited but metabolically mature environment, including *A. muciniphila*, which utilises host-derived mucin glycans as its main carbon source and engages in cross-feeding with butyrate-producing *Clostridia*. This microbial configuration is typically associated with improved gut barrier function and higher production of short-chain fatty acids. Moreover, the increase in the abundance of *A. muciniphila* reflects its adaptation to polyphenol-enriched, low-nutrient conditions, where it degrades mucins to produce SCFAs that support gut barrier integrity and reduce inflammation [44]. Importantly, viable *A. muciniphila* has been demonstrated to play a key role in reversing metabolic disorders induced by a high-fat diet, including reducing fat mass gain, metabolic endotoxemia, and insulin resistance. These effects are strictly dependent on bacterial viability and are associated with increased intestinal levels of endocannabinoids that control inflammation and gut barrier function [45].

Fermentation thus shifted dynamics toward *Akkermansia*-driven homeostasis. In contrast, the non-fermented pomace retained a high proportion of intact dietary polysaccharides and fibres, providing a rich substrate for *B. fragilis*, a species equipped with numerous polysaccharide utilisation loci (PULs) that enable the breakdown of plant cell wall components, such as pectins and hemicelluloses [44]. While *B. fragilis* plays a key role in fibre degradation, its dominance at the expense of mucin-degrading species may indicate a less diverse and potentially metabolically less stable ecosystem compared to trophic networks involving *Akkermansia* and butyrogenic *Clostridia* [45]. This contrast underscores that fermentation not only alters substrate composition but also redefines trophic relationships within the microbiota, steering it toward a functionally more balanced profile associated with host metabolic health. Its dominance in the control group indicates a metabolism oriented toward the saccharolytic degradation of plant-derived glycans, leading mainly to acetate, succinate, and propionate formation. While this activity supports carbohydrate fermentation, it lacks the mucin-degrading and cross-feeding capacity observed in the fermented group. Together, these findings suggest that pre-fermentation of apple pomace redirected the gut microbial metabolism from a fibre-driven system dominated by *Bacteroides* toward a mucin- and SCFA-oriented network involving *Akkermansia* and butyrogenic *Clostridia*, consistent with a more functionally balanced microbiome configuration associated with health [46,47,48,49,50].

*Pseudomonadota* remained at a high level in the control samples supplemented by non-fermented apple pomace, but their abundance declined significantly in the research samples with fermented apple pomace (Figure 2B and Figure S1D in the Supplementary Materials). Elevated abundance of *Pseudomonadota* can be associated with dysbiosis in the gut. However, the suppression of this phylum is frequently reported in studies involving fermented foods and by-products rich in organic acids and biotransformed polyphenols [51]. The reduced abundance of *Klebsiella*, *Serratia*, and *Raoultella* suggests that fermentation not only promotes beneficial taxa but also helps stabilise the microbial ecosystem by reducing the abundance of potential pathobionts [52].

It is also worth noting the reduction in the relative abundance of *Phocaeicola dorei*, with the simultaneous increase in *Parabacteroides distasonis*, *Bacteroides thetaiotaomicron*, and *Bacteroides faecis* (Figure 2B and Figure S1B in the Supplementary Materials). This clearly demonstrates the prebiotic effect of metabolites and residual fibres obtained during fermentation, which stimulated fibre-degrading and SCFA-producing bacteria that influence gut barrier function and show anti-inflammatory activity [53,54,55]. *P. dorei* supports liver and gut health by producing SCFAs, modulating bile acid metabolism, and regulating immune responses. A decrease in its abundance may negatively influence these effects [56]. *P. distasonis* exhibits anti-inflammatory and immunomodulatory properties and may promote liver regeneration via β-hydroxybutyrate and STAT3 signalling. However, its excessive abundance may disturb microbial balance [55]. *B. thetaiotaomicron* can degrade complex polysaccharides into more digestible forms. Moreover, by producing SCFAs it can support gut barrier function and immune modulation. However, excess abundance can exacerbate inflammation, while low levels reduce metabolic and immune homeostasis [54]. *B. faecis* has been found to protect the gut from inflammation by enhancing barrier integrity and promoting Treg/Th17 balance in experimental colitis models [57].

While fermented apple pomace promoted the growth of beneficial fibre-degrading bacteria, a higher abundance of potentially pro-inflammatory taxa, including *Desulfovibrionaceae*, *Dethiosulfovibrionaceae*, and *Fusobacterium varium*, was observed. These bacteria are well-known producers of hydrogen sulphide and other metabolites, which can impair gut barrier function and promote local inflammation [58,59]. Increased abundance may impact the availability of fermentation-derived substrates that these taxa can use, highlighting that fermented dietary products can simultaneously stimulate both beneficial and potentially deleterious gut microbes. *Desulfovibrionaceae* and *Dethiosulfovibrionaceae* generate H_2_S, which at moderate levels aids energy metabolism but in excess inhibits butyrate producers and impairs barrier function [60]. High concentrations of H_2_S inhibit butyrate oxidation in colonocytes, leading to epithelial energy starvation, increase intestinal barrier permeability by disrupting tight junctions, and promote inflammation [59,61]. Microbial pathways involved in colonic sulphur metabolism are complex, with sulphate-reducing bacteria (SRB), such as *Desulfovibrio*, being common colonic inhabitants that utilise both inorganic (sulphates, sulphites) and organic (dietary amino acids, host mucins) sulphur sources [61]. Analysis of *Desulfovibrio* strains isolated from individuals with colitis has demonstrated that biomass accumulation and hydrogen sulphide production are key factors in the development of bowel inflammation, including ulcerative colitis [62]. *F. varium* similarly competes for substrates, suppressing SCFAs while promoting inflammation, as seen in colitis models [63].

The non-fermented apple pomace in control samples did not induce a similar increase in the discussed taxa. This suggests that fermentation influences the substrate profile, selectively favouring certain microorganisms. These findings should draw attention to both the positive and negative effects on microbial communities when designing functional fermented foods [64]. Such observations are crucial from the perspective of functional food design. They emphasise that dietary interventions rarely lead to unequivocally positive changes, but instead often induce multidirectional, complex effects in an ecosystem as intricate as the gut microbiota. In this study, the increase in potentially pro-inflammatory taxa was balanced by strong beneficial signals, such as a rise in *Akkermansia* and SCFA production and a decrease in *Pseudomonadota* (including pathobionts from the genus *Klebsiella*). Therefore, assessing the overall impact on host health requires a holistic view—the beneficial SCFA profile and the increase in barrier-protective bacteria may outweigh the negative effect of increased H_2_S producers.

The observed differences in gut microbiota composition following supplementation with fermented and non-fermented preparations highlight the fundamental role of substrate quality and transformation in shaping microbial ecology. Apple pomace fermented by *K. barnettii* D1 acted as a modulated source of amino acids and peptides, which are more bioavailable. Such pre-digestion likely met the metabolic demands of diverse gut taxa, supporting balanced growth of saccharolytic and butyrate-producing bacteria and favouring the growth of more demanding bacterial species. In contrast, the non-fermented pomace provided less accessible plant proteins and intact polysaccharide structures, selecting for cellulolytic and proteolytic microorganisms able to degrade raw cellulose and complex fibres. Thus, the microbial community responded not only to the type of substrate but also to its biochemical accessibility, with fermentation effectively shifting the metabolic landscape toward a more functionally stable and health-associated configuration [65].

### 2.3. Metabolomic Analysis of Gut Microbiota Across the 4-Week Period of Supplementation with Fermented and Unfermented Apple Pomace

Metabolomic analysis enables identification of low-molecular-weight metabolites, reflecting functional changes within complex biological systems and complements sequencing data by revealing bioactive compounds produced and transformed by microbial communities [66]. Among the various “omics” techniques, metabolomics is the most closely related to phenotype. Microbial metabolites are known to be direct regulators of biological processes [67]. In the context of the human microbiome, metabolomic analysis provides critical insight into the functioning of microbial communities. It strongly supports genomic and microbiome sequence information by providing information on bioactive compounds produced and consumed by the microbial community. Simultaneous changes in the metabolomic profile of the intestinal environment usually result from the composition and activity of gut microbiota [66]. Many factors can affect these phenomena, such as dietary habits, drug administration, issues of genetic origin, and diseases. These factors strongly influence the human microbiota and the metabolites they produce. Metabolomic profiling enables the measurement of such alterations in the metabolism and the identification of specific metabolites associated with such disruptions [68]. This approach enabled the identification and comparison of the dominant chemical categories of metabolites that either increased or decreased as a result of fermentation. Identification of the same metabolite as significant in both positive and negative ionisation modes provided additional confirmation of its biological relevance.

**Figure 3:**
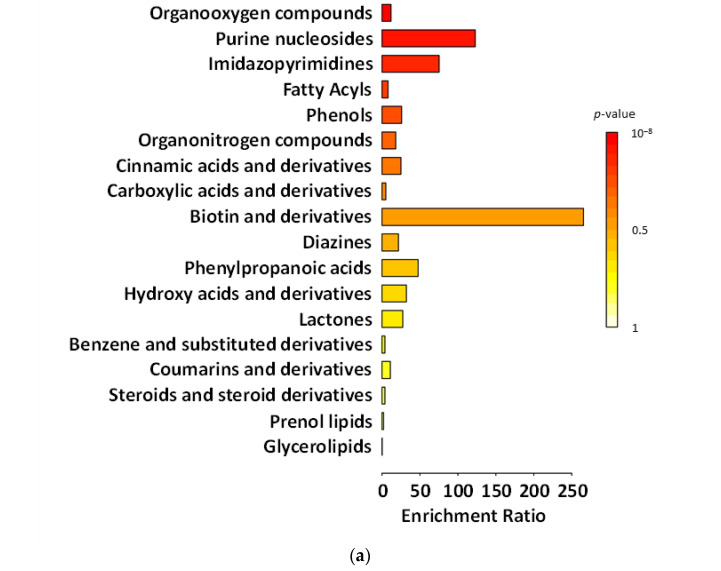

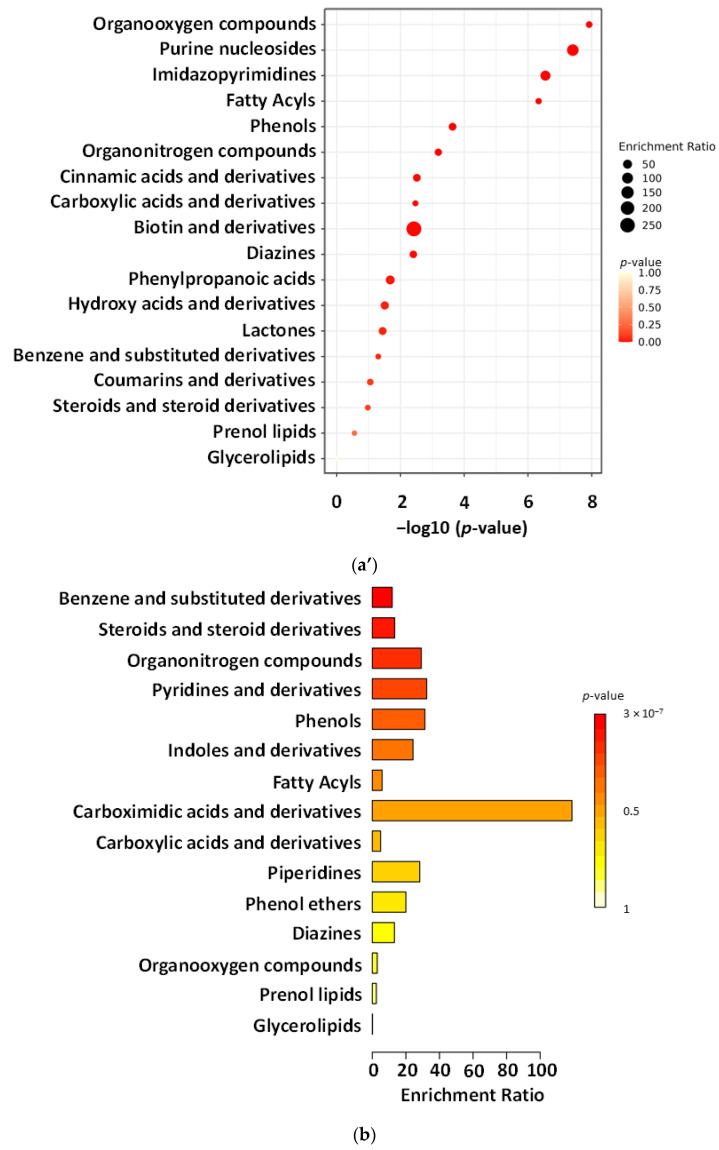

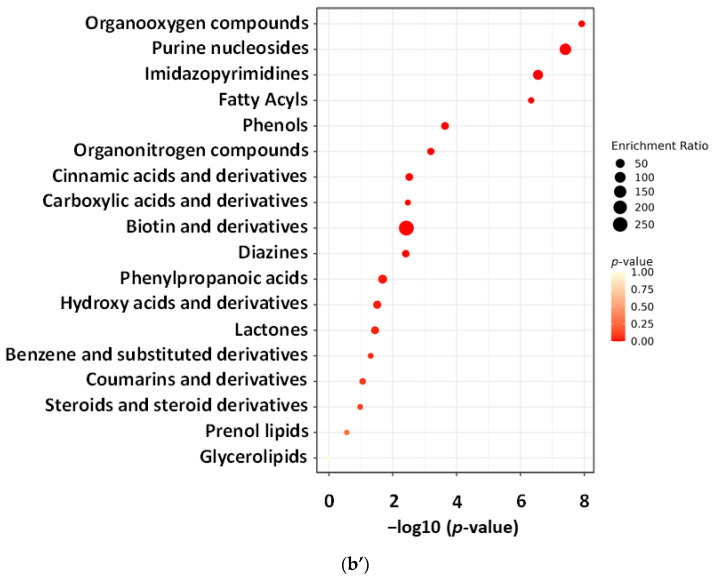
Chemical main-class enrichment analysis for metabolites with elevated abundance in gut microbiota samples from models supplemented with the fermented apple pomace (**a**,**a’**) and non-fermented apple pomace as a control sample (**b**,**b’**) during the first week of the main experimental phase (‘T1’). Bar plot showing the top enriched metabolite classes based on the enrichment ratio and *p*-value (colour-coded). Bubble plot representing the same classes, with dot size indicating the enrichment ratio and colour reflecting statistical significance (*p*-value).

**Figure 4:**
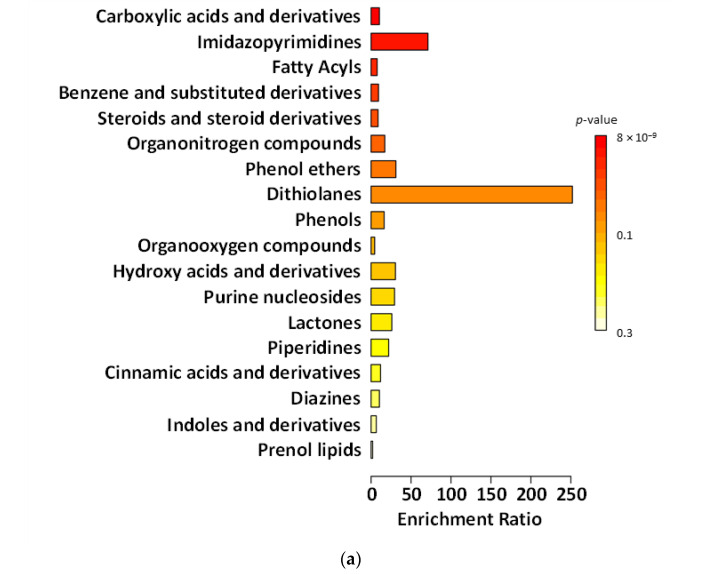

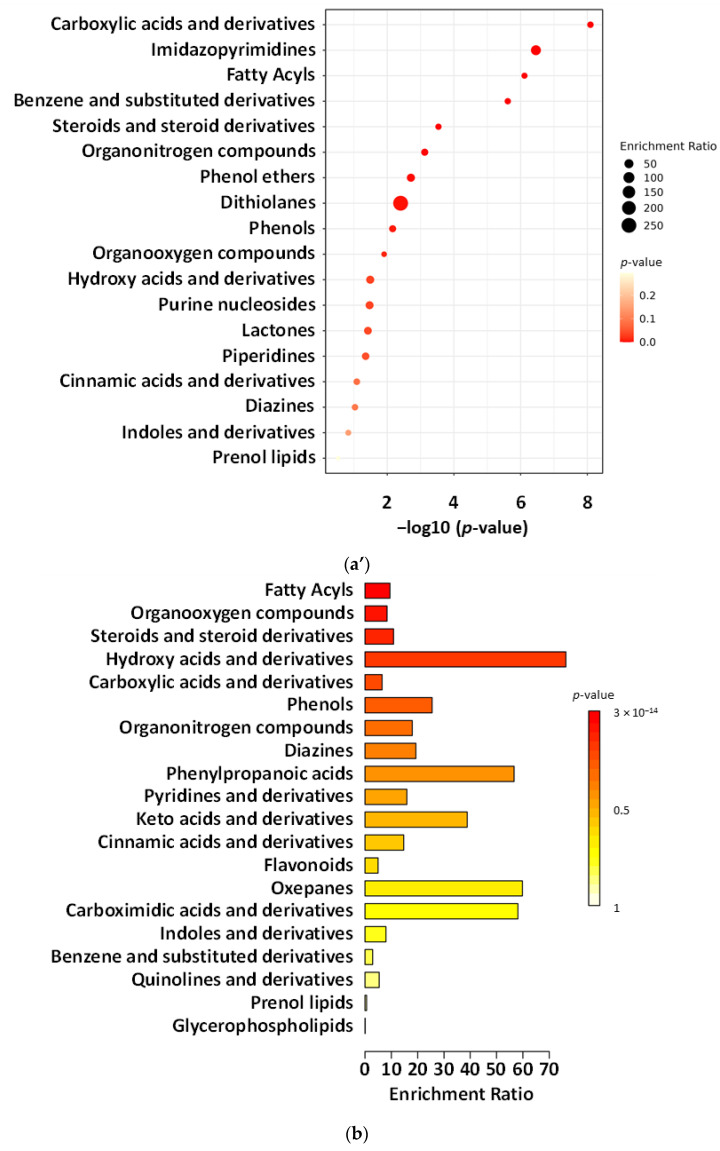

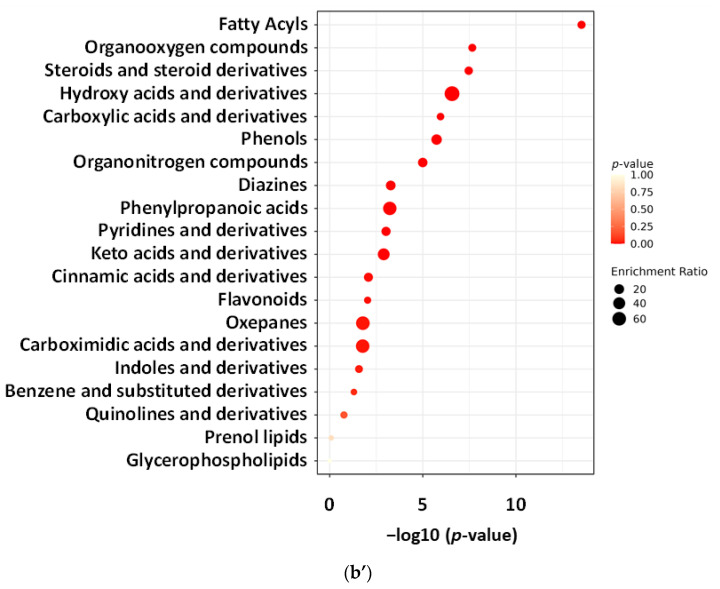
Chemical main-class enrichment analysis for metabolites with elevated abundance in gut microbiota samples from models supplemented with the fermented apple pomace (**a**,**a’**) and non-fermented apple pomace as a control sample (**b**,**b’**) during the second week of the main experimental phase (‘T2’). Bar plot showing the top enriched metabolite classes based on the enrichment ratio and *p*-value (colour-coded). Bubble plot representing the same classes, with dot size indicating the enrichment ratio and colour reflecting statistical significance (*p*-value).

**Figure 5:**
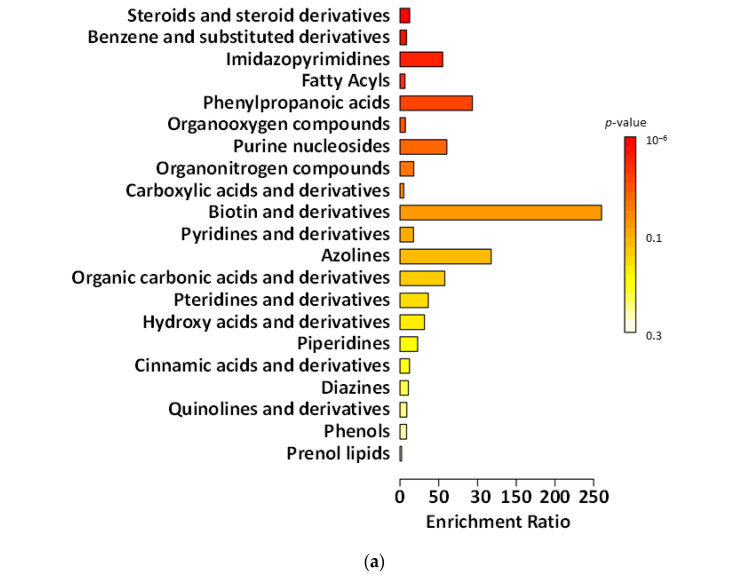

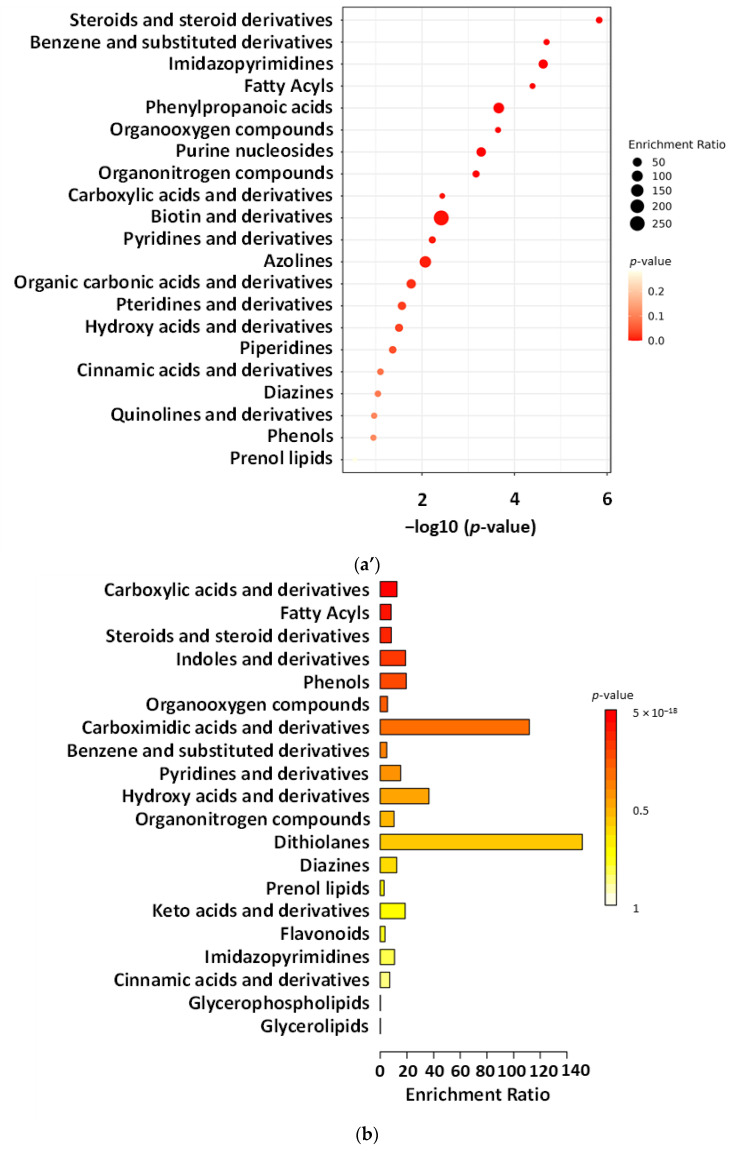

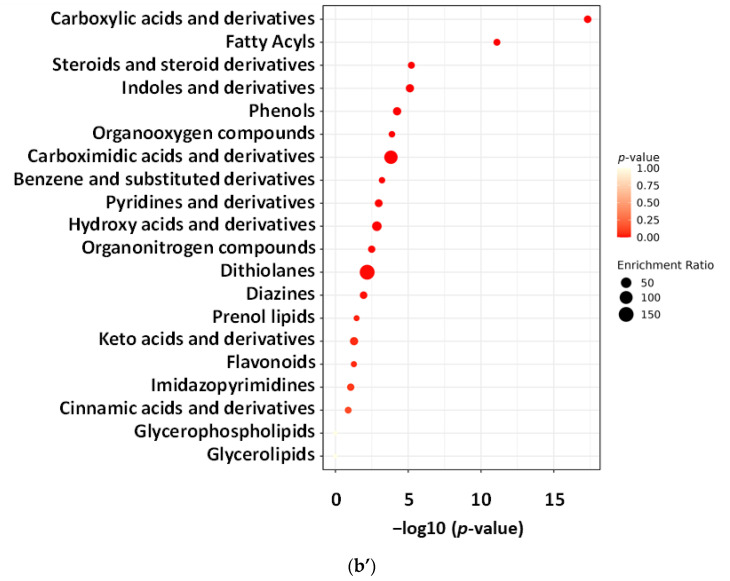
Chemical main-class enrichment analysis for metabolites with elevated abundance in gut microbiota samples from models supplemented with the fermented apple pomace (**a**,**a’**) and non-fermented apple pomace as a control sample (**b**,**b’**) during the last day of the main experimental phase (‘T5’). Bar plot showing the top enriched metabolite classes based on the enrichment ratio and *p*-value (colour-coded). Bubble plot representing the same classes, with dot size indicating the enrichment ratio and colour reflecting statistical significance (*p*-value).

**Figure 6:**
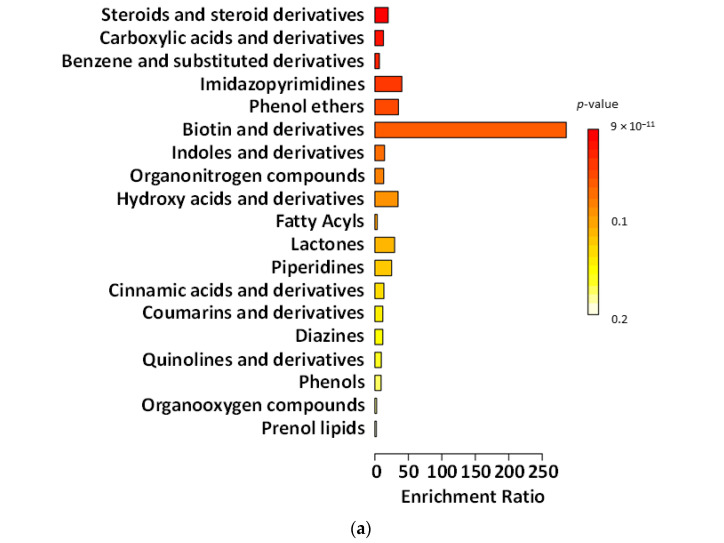

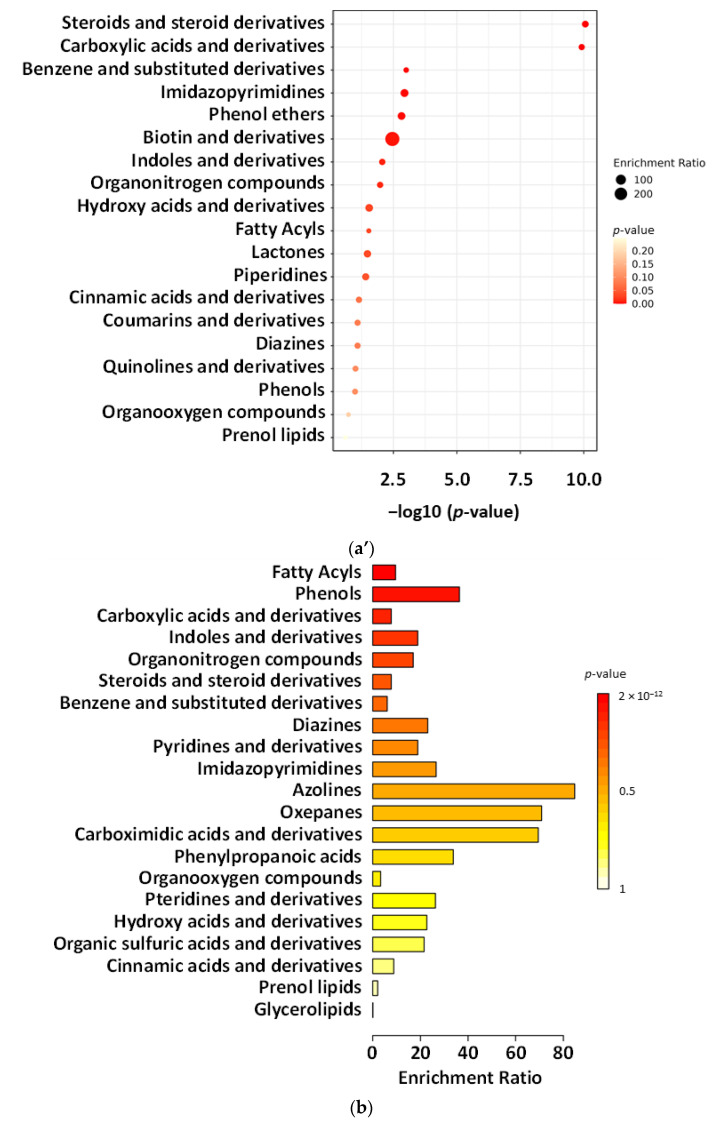

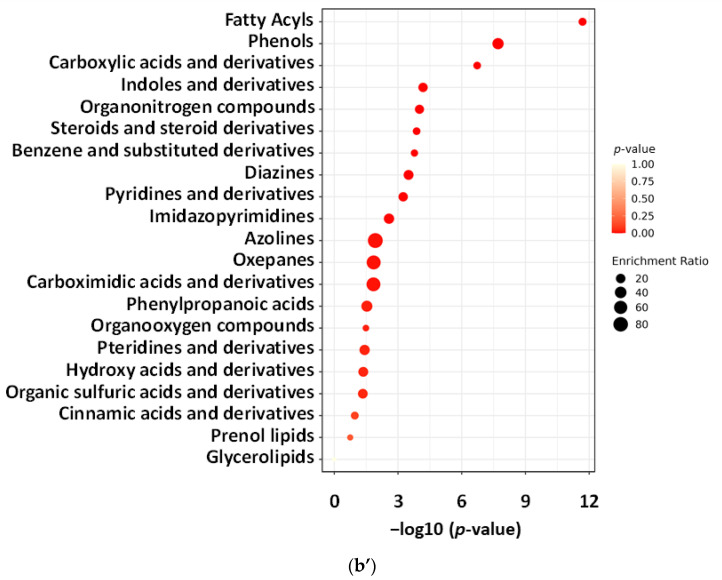
Chemical main-class enrichment analysis for metabolites with elevated abundance in gut microbiota samples from models supplemented with the fermented apple pomace (**a**,**a’**) and non-fermented apple pomace as a control sample (**b**,**b’**) during the silencing phase (‘S’). Bar plot showing the top enriched metabolite classes based on the enrichment ratio and *p*-value (colour-coded). Bubble plot representing the same classes, with dot size indicating the enrichment ratio and colour reflecting statistical significance (*p*-value).

Figure 3, Figure 4, Figure 5 and Figure 6 present chemical main-class enrichment analysis for metabolites elevated in gut microbiota samples from the first week (‘T1’), second week (‘T2’), and last day (‘T5’) of the main experimental phase of supplementation with fermented and non-fermented apple pomace, as well as on the last day of the silencing phase (‘S’). The bar plots show the top enriched metabolite classes based on the enrichment ratio and *p*-value (colour-coded). Bubble plots represent the same classes, with dot size indicating the enrichment ratio and colour reflecting statistical significance (*p*-value) (Figure 3, Figure 4, Figure 5 and Figure 6).

In the experiment supplemented with fermented apple pomace, the levels of compounds classified as phenols gradually declined during both the supplementation period and the silencing phase (‘S’). This indicates that the fermented apple pomace did not stimulate but rather reduced phenol production. In the gut, simple phenols (e.g., phenol, *p*-cresol) are typical end products of proteolytic fermentation of aromatic amino acids, especially tyrosine, produced by *Clostridium* spp. and related *Firmicutes* [69]. A sustained decrease in these metabolites suggests that supplementation may have shifted the microbiota from a proteolytic metabolism toward a more saccharolytic profile, in which short-chain fatty acids (SCFAs) dominated instead of toxic phenolic by-products. This interpretation is supported by the observed rise in *Akkermansia* and butyrate-producing *Clostridia*, taxa commonly associated with enhanced SCFA output and reduced proteolytic metabolite formation [69]. In contrast, in the experiment supplemented with non-fermented apple pomace, phenolic levels constantly increased, indicating that the untreated apple pomace itself added phenols or acted as a matrix for their biosynthesis. We also observed biotin production by *B. cellulosilyticus*, which can be utilised by *A. muciniphila*, supporting their metabolic coexistence within the gut ecosystem.

The abundance of benzene and substituted derivatives contrasted with the changes observed for phenols. Supplementation with fermented apple pomace increased the abundance or benzene and substituted derivatives, which remained relatively constant even in the silencing phase (‘S’). This growth pattern affects the microbiota by favouring microorganisms capable of producing aromatic acids from dietary and host-derived substrates. Many *Clostridia* generate such compounds through the fermentation of phenylalanine and tyrosine [70]. In addition, the increase in *Akkermansia* and SCFA-producing *Clostridia* may have supported cross-feeding networks that stabilise aromatic metabolism beyond the active supplementation phase [71,72,73]. By contrast, in the control supplemented with non-fermented apple pomace, the levels of benzene derivatives were consistently high, but did not change significantly over time, suggesting a steady-state production of these metabolites by the existing microbiota. This may indicate that the non-fermented apple pomace had no significant effect.

The research and control groups also showed differences in metabolite classes, such as fatty acyls and steroids (and steroid derivatives). The steroids and steroid derivatives detected were derived from bile acids. Gut bacteria play a crucial role in bile acid transformation. *Firmicutes*, especially *Clostridia*, perform dehydroxylation of primary bile acids to produce secondary bile acids, such as deoxycholic acid and lithocholic acid [74]. In the experiment supplemented with fermented apple pomace, the increase in *Clostridia* may have enhanced this conversion process. Thus, as the fermented group progressed, one would expect higher levels of secondary bile acid derivatives, which would be detected in the steroid class. Indeed, the steroid metabolite levels rose even after the supplementation period. This suggests that more primary bile acids present in the gut were being deconjugated and transformed by the expanding gut microbes. *Akkermansia* may also have contributed to this process, as it possesses bile salt hydrolase activity to deconjugate bile acids, making them available for further conversion by *Clostridia* [75]. Such changes have functional implications. Secondary bile acids can activate host receptors affecting lipid and glucose metabolism and also inhibit the growth of pathogens [76,77]. In the non-fermented group, the increase in steroids and steroid derivatives during supplementation is likely to have been related to the intake of apple pomace-derived phytosterols, such as β-sitosterol and triterpenic acids (especially ursolic and oleanolic acid) found in apple skin and seeds. Apple pomace is known to contain significant amounts of these compounds, which can be detected in untargeted metabolomic assays and may overlap with host-derived bile acid signals. Their decrease in the silencing phase supports the conclusion that the increase in these compounds was not microbial in origin [78,79,80].

Fatty acyls were also qualitatively measured in the experiment. As the whole system operated on an artificial intestine, compounds classified as fatty acyls most likely derived from the apple-pomace matrix (peel/seed waxes, long-chain fatty acids) [81,82]. In the non-fermented control, fatty acyl levels increased steadily across supplementation and washout, consistent with the slow release of hydrophobic residues and limited microbial degradation in a community lacking *Akkermansia* and with a decrease in *Firmicutes*. On the other hand, in the experiment with fermented apple pomace, the level of fatty acyls was more stable. Fermentation conducted by *K. barnettii* D1 influenced the pomace matrix by partially degrading and modifying more complex components, thereby reducing the release of hydrophobic precursors. This aligns with the documented enzymatic activity of *K. barnettii* and its ability to reshape the metabolite profile of plant-based substrates during low-temperature fermentation [21,83]. These results suggest that the type of pomace (fermented vs. non-fermented), rather than external lipid metabolism, determined the fatty-acyl pattern. Fermentation before supplementation helped to keep the levels more stable and prevented their late increase.

### 2.4. SCFA Analysis Produced by Gut Microbiota During 4-Week Supplementation with Fermented and Unfermented Apple Pomace

Short-chain fatty acids (SCFAs), primarily acetate, propionate and butyrate, are key microbial metabolites for maintaining intestinal homeostasis [84,85]. There are three main SCFAs: acetic acid, propionic acid, and butyric acid. Their concentration in the intestine ranges from 20 mM to 140 mM, and their typical ratio is 60–65:20:15–20 [86,87].

Acetate production is a typical fermentation end product for various genera of bacteria. The typical producer of this metabolite is *A. muciniphila* [88]. However, there are also uneven microorganisms that synthesise this SCFA. For instance, despite being primarily butyrate-producing bacteria, *Ruminococcus* spp. are also acetate producers. Similarly, *Prevotella* spp., which mainly produce propionate, are able to produce acetate [87,89]. The production of acetate by gut microbiota provides health benefits such as protection against obesity and weight gain and may influence metabolic disorders as well as immune modulation [90,91,92,93].

Acetic acid is also a fundamental substrate for butyrate-producing bacteria, including *Eubacterium rectale*, *Roseburia* spp., and *Faecalibacterium prausnatzii* [94,95]. Different metabolic pathways are used by these bacteria for the production of butyrate. One occurs via the acetate CoA-transferase pathway, which converts acetate into butyrate. Butyrate is associated with the integrity of the intestinal epithelium. It is the substrate preferred by colonocytes as an energy source. It is also associated with cardiovascular health, immune regulation, and appetite control [95]. Supplementation with fermented apple pomace did not lead to a visible increase in the concentration of this metabolite. Butyric acid levels remained stable, consistent with its physiological concentration. In the control samples, 4-week administration of untreated apple pomace significantly decreased the concentration of butyric acid.

Another important SCFA is propionic acid and its salts. Two pathways are known for the formation of propionate from sugar fermentation by gut bacteria. One is the succinate pathway processing hexose and pentose sugars. The succinate pathway is found mainly in *Bacteroidetes* and in the *Negativicutes* class of *Firmicutes* [96]. This is the major route of propionate formation from dietary carbohydrates driven by *Bacteroidetes*. The other mechanism involves the propanediol pathway, in which fucose and rhamnose are metabolised. In this pathway, the formation of propionate and propanol is conducted by dominant gut commensal bacteria belonging to *Lachnospiraceae*, including *Roseburia* spp. Interestingly, propionate and butyrate can also be synthesised from peptide and amino acid fermentation. However, it is estimated that less than 1% of the bacteria present in the large intestine is able to perform this fermentation process [97,98]. The concentration of propionic acid during supplementation with fermented apple pomace was, in general, constant. In the control experiment, supplementation with untreated apple pomace induced a significant increase in propionic acid in the ascending colon. Its concentration was higher only during the silencing phase (‘S’).

**Figure 7:**
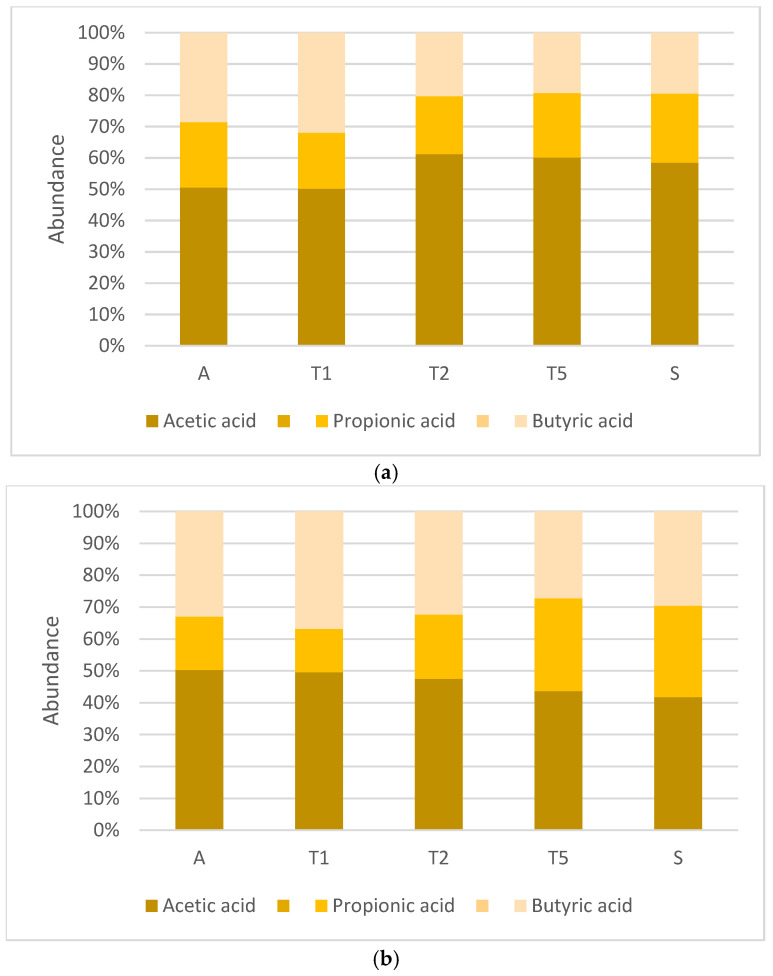
Relative abundance of short-chain fatty acids in ascending colon samples from the main experiment (**a**) and control (**b**). Stacked bars show the percentages of acetic acid (brown), propionic acid (yellow), and butyric acid (beige) across time points: ‘A’—last day of the adaptation phase of the intestinal microbiota before the main phase of the experiment; ‘T1’—first week of the main phase of the experiment (supplementation with fermented apple pomace in the research line and supplementation with non-fermented apple pomace in the control line); ‘T2’—second week of the main phase of the experiment; ‘T5’—the last day of the main phase of the experiment before the silencing phase; ‘S’—last day of the silencing phase of the experiment.

Figure 7 presents the results of the analysis of the SCFA ratio during the main experiment and in the control. During 4-week administration of fermented apple pomace, the ratio of acetic acid:propionic acid:butyric acid increased from around 50:18:32 to 70:15:15. Before supplementation and in the first week of supplementation (‘T1’), an excess of butyric acid was observed. However, in the fourth week of the main stage of the experiment, all SCFAs normalised, oscillating around the physiological ratio for these compounds described in the literature [99,100,101]. Within the silencing phase, without supplementation, the level of acetic acid decreased and the proportions of the three SCFAs were within the reference range. In the control experiment, the acetic acid:propionic acid:butyric acid ratio was found to be significantly beyond the range defined in the literature. Although total SCFA production was slightly higher than in the main experiment, the altered proportions of individual SCFAs may suggest changes in microbial metabolic activity and potentially reflect shifts in the diversity or stability of the large intestinal microbiota.

The most significant observation was the increased proportion of butyric acid accompanied by a reduced level of propionic acid. Butyric acid plays a key role in maintaining intestinal barrier integrity, whereas propionic acid is involved in immune regulation, metabolic processes, and protection against inflammation-associated intestinal dysfunction. An imbalance between these short-chain fatty acids has been associated with disorders such as inflammatory bowel disease [102,103]. The differences between the main experiment and control group may result from the difference in the complexity of the fermented and untreated apple pomace. During fermentation, complex compounds such as dietary fibre are broken down into digestible forms preferred by the human organism. Thus, the fermented apple pomace could provide simple compounds, which were absorbed in the large intestine quickly and did not provide substrates for microbes. In contrast, the untreated apple pomace in the control sample delivered more dietary fibre, which served as a substrate for further microbial activity, resulting in higher levels of SCFAs. On the other hand, the apple pomace without microbial processing contained fewer easily digestible compounds, such as vitamins and minerals [104].

## 3. Materials and Methods

### 3.1. Raw Material and Microorganisms Used in the Experiment

#### 3.1.1. Preparation of Apple Pomace

Apples of the “Ligol” variety (previously characterised by Yoon and coworkers [105]) were washed and dried. Apple pomace was prepared in the laboratory by pressing the fruits using a slow-speed juicer (Gallet model HS 703, Prague, Czech Republic). The pomace was subsequently passed through the machine again to remove the rest of the juice. The pressed apple mass was suspended in a ratio of 3:1 in water, and the final suspension was pasteurised at a temperature of 78 °C for 20 min.

#### 3.1.2. Yeast Strains and Cultivation

Three environmental non-*Saccharomyces* yeast isolates previously deposited in the NCBI database were used in the experiments: *Kazachstania barnettii* D1 from pickled beetroot (accession no. OQ275007.1), *Hanseniaspora uvarum* D9 from grape (accession no. OQ275152.1), and *Wickerhamomyces anomalus* D11 from wheat flour (accession no. OQ275025.1).

The yeasts were initially characterised by Liszkowska and coworkers [106] and were proven to valorise apple pomace by forming significant amounts of volatile organic compounds at 15 °C [21].

The strains were cultured in 100 mL of wort broth (Merck, Darmstadt, Germany) at 15 °C for 7 days. Yeast growth was assessed by measuring turbidity with a densitometer (DEN-1B, Grant Instruments (Cambridge) Ltd., Shepreth, UK), which provides a direct readout in McFarland units. The culture reached ~8 °McF. A low temperature was implemented during the activation stage to maintain constant activity in the tested strains throughout the process.

### 3.2. Liquid-State Fermentation of Apple Pomace

After activating the yeast strains, 100 mL of each apple pomace prepared according to the method described in Section 3.1.1 was inoculated with 10% inoculum of each strain (optical density of the suspension ~8 °McF). Fermentation was performed at 15 °C for five days. Details of the fermentation procedure are provided by Liszkowska and coworkers [21].

### 3.3. INFOGEST Static In Vitro Simulation of Gastrointestinal Digestion

The viability rates of the three tested yeast strains under conditions of gastrointestinal passage were evaluated according to the INFOGEST static in vitro simulation of gastrointestinal digestion protocol [107].

INFOGEST is a static in vitro digestion method that simulates gastrointestinal conditions, where samples are exposed to predefined gastric and intestinal environments, including bile salts and digestive enzymes. This approach enables reliable assessment of the proportion of the yeast cell population that was able to maintain activity under gastrointestinal stress. The viability of cells exposed to the gastrointestinal passage was calculated in the control sample (before conducting the process) and, after each stage in the simulation, expressed in log(CFU/mL).

### 3.4. Simulator of the Human Intestinal Microbial Ecosystem (SHIME^®^)

The prebiotic effect of the fermented apple pomace and the biological activity of the cold-adapted yeast used for fermentation were assessed in a Simulator of the Human Intestinal Microbial Ecosystem (SHIME^®^). This system enables the simulation and maintenance of human gastrointestinal microbial diversity in vitro. In this system, the pH, residence time, and temperature are controlled by software. The SHIME^®^ consists of five vessels simulating the stomach, the duodenum (or small intestine), and the ascending, transverse, and descending colon. The system is kept free of oxygen by providing nitrogen input [108]. At the beginning of the experiment, three colon vessels were inoculated with faeces diluted in model medium (soluble starch, mucin, yeast extract, arabinogalactan, xylan, peptone tryptone, L-cysteine hydrochloride, glucose, and pectin). The faecal inoculum was obtained from a healthy adult donor who maintained a balanced diet for 6 months prior to the experiment, without excluding any food components. During this period, the donor reported no consumption of probiotic or prebiotic supplements and did not take any prescription medications or antibiotics.

Inoculation was repeated three times over six days, occurring every two days. After this time, the experimental protocol included a two-week control period after the inoculation of the colon to allow the adaptation of the microbial community to physicochemical and nutritional conditions prevailing in different parts of the colon [24] and also to stabilise the microbial community [109,110]. During this period, the feed consisted of model medium introduced to the system three times a day.

After two weeks of stabilisation, the protocol was followed by four weeks of treatment with apple pomace fermented at 15 °C by cold-adapted yeast. The test sample was entered once a day. The two remaining feed portions consisted of model medium. Unfermented apple pomace was used as a control.

After four weeks in the main stage of the experiment, the fermented apple pomace was replaced by the model medium, and the viability of yeast able to survive under the gastrointestinal passage conditions was assessed by assessing growth by the pour plate method.

During the experiment, samples were collected from the ascending colon (from research and control lines) and marked as follows:

- ‘A’—the last day of the adaptation phase of the intestinal microbiota before the main phase of the experiment;
- ‘T1’—the first week of the main phase of the experiment (supplementation with fermented apple pomace in the research line and with non-fermented apple pomace in the control line);
- ‘T2’—the second week of the main phase of the experiment;
- ‘T5’—the last day of the main phase of the experiment before the silencing phase;
- ‘S’—the last day of the silencing phase of the experiment.

### 3.5. Analysis of Short-Chain Fatty Acids by High-Performance Liquid Chromatography Coupled with a PDA Detector

Protein compounds were removed from the samples collected from the ascending colon of the gastrointestinal model using the Carrez method. First, the samples were centrifugated at 4000 rpm for 10 min. Then, 3.5 mL of supernatant was collected from each sample and mixed with 0.2 mL of Carrez I solution. After 2 min, 0.2 mL of Carrez II solution was added, followed by 3.1 mL of distilled water. The samples were centrifugated again at 4000 rpm for 10 min. The obtained supernatant was filtered using syringe filters with a pore size of 0.22 µm.

Samples were analysed using high-performance liquid chromatography coupled with a PDA detector. For this purpose, 20 µL of each sample was eluted with sulfuric acid at a rate of 0.5 mL/min. The time of the analysis was 60 min. The column used was a Repromer H, 9 µm, y = −0.0098x + 21,778, R^2^ = 0.9829. The temperature of the oven was held at 60 °C. The detector used was a photo-diode array.

### 3.6. Metagenomic Analysis

#### 3.6.1. DNA Isolation and Sequencing of Metagenomes

Genomic DNA was isolated from the SHIME samples. NGS libraries were prepared using the DNA Library Prep Set. The isolated genomic DNA was sequenced on an Illumina NovaSeq X sequencing platform (Illumina, San Diego, CA, USA). Between 36,572,742 and 17,042,342 pairs of 150 bp reads were generated per library. NGS library preparation and sequencing was performed at weSEQ.IT. The raw reads were deposited in the NCBI database under the BioProject accession PRJNA1381616 and BioSample accessions SAMN54116487–SAMN54116492.

#### 3.6.2. Metagenomic Binning

Metagenome-assembled genomes (MAGs) were generated for each sample using the metaWRAP pipeline (v. 1.3.2; [111]), employing FastQC to control the quality of sequencing reads [112], Trim Galore! (v. 0.5.0; [113]) and Cutadapt (v. 1.18; [114]) for quality and adapter trimming of the sequencing reads, BMTagger for removing human (hg38) reads [115], SPAdes in metaspades mode for assembling of metagenomes (v. 3.13.0; [116]), QUAST for quality assessment of the assemblies (v. 4.1; [117]), metaBAT 2 (v. 2.12.1; [118], MaxBin 2.0 (v. 2.2.6; [119]) and CONCOCT (v. 1.0.0; [120]) for binning of assemblies, and CheckM for metagenomes evaluation [121]. Quality filtering of MAGs was set at ≥70% for completeness and ≤5% for contamination. Consolidated MAGs from all samples in the study were dereplicated using dRep (v. 3.5.0; [122]) at an average nucleotide identity (ANI) threshold of 95% with default options. In total, 64 species-level representative genome bins (SRGs) were obtained. The taxonomy of the dereplicated metagenomes was determined using the “BIN_CLASSIFICATION” module of MetaWRAP and the NCBI_nt _24012025 database. CoverM (v. 0.4.0; [123]) was used to calculate the percentage abundance of dereplicated metagenomes for each NGS library.

Processing of MAGs information was performed using custom-made scripts written in Perl v. 5.26.2 or Python v. 3.7.9. The calculations mentioned in this paper were performed using the BlueOcean computational cluster, which is part of the Łódź University of Technology Computing & Information Services Centre infrastructure.

### 3.7. Metabolomic Analysis

#### 3.7.1. Analysis of Gut Microbiota Samples from Models Supplemented with Fermented and Non-Fermented Preparations at Each Time Point

Extracts for LCMS were prepared by adding acetone (900 μL) to the post-culture medium (300 μL), with mixing on a vortex shaker for 1 min. Then, the sample was incubated at −20 °C for 60 min and centrifuged for 5 min (4 °C, 12,000× *g*). The supernatant was transferred to a previously weighed Eppendorf vial. The content of the vial was dried and evaporated under a vacuum using a speed vac (2 mbar) for approximately 15 h. The dry extract was weighed and then dissolved in methanol (0.13 mg per 80 μL of methanol). The dissolved extract was centrifuged (4 °C, 12,000× *g*, 5 min) and transferred to an HPLC vial.

#### 3.7.2. UHPLC-QToF-MS and MS/MS Analysis of Extracts

Mass spectrometry–liquid chromatography analyses were performed on a Bruker Elute UHPLC system operated by Hystar 3.3 software and a Bruker Impact II ultrahigh resolution (60,000+) mass spectrometer (Bruker Daltonik GmbH, Bremen, Germany) ESI QTOF-MS equipped with Data Analysis 4.2 (Bruker Daltonik GmbH, Bremen, Germany), TASQ (ver. 2022b) and Metaboscape (ver. 2022b). The column used for AutoMSMS measurements was a reverse-phase Bruker Intensity Solo C18 with 2 µm particles and dimensions of 2.1 × 50 mm. Eluent A was water with 0.1% HCOOH (Sigma-Aldrich, St. Louis, MO, USA) and eluent B was acetonitrile (Sigma-Aldrich, St. Louis, MO, USA) with 0.1% HCOOH. First, 5 μL of extract was loaded on the column at a flow rate of 200 μL/min, using 4% B. The percentage of Eluent B was changed over time as follows: 0 min—1%; 0.56 min—1% B; 4.72 min—99%; 5.56 min—99%; 5.6 min—1%; and 9.45 min—1%. Solvent flow was 450 μL/min. The column was held at 40 °C. The column exit was connected to the ESI source. AutoMSMS measurements were conducted in both positive and negative ion modes in the *m*/*z* range 50–1200. Collision-Induced Dissociation (CID) was performed with the following settings: an absolute area threshold of 5000 counts, active exclusion after 2 spectra, and release after 0.3 min. The isolation mass settings were as follows: for *m*/*z* = 100, the width was 4; for *m*/*z* = 300, the width was 5; for *m*/*z* = 500, the width was 6; and for *m*/*z* = 1000, the width was 8. The collision energy was set to 30 eV. Internal calibration on 10 mM sodium formate (Sigma-Aldrich, St. Louis, MO, USA; in 1:1 = water:isopropanol *V*/*V*) ions was performed automatically in Metaboscape using a syringe pump at an infusion flow rate of 1.5 µL/min in high precision calibration (HPC) mode. The untargeted annotations were performed in Metaboscape (ver. 2022b) with a criterion of mass deviation (Δ*m*/*z*) under 2 ppm and mSigma value under 20 as the maximum acceptable deviations of the mass of the compound and the isotopic pattern, respectively [39,40]. All the molecular formulas were obtained using the Smart Formula tool and the C, H, N, O, P, S, Cl, Br, I, and F elements. MSMS spectra were automatically matched against MSMS libraries: the Bruker HMDB 2.0 library (spectral data with retention times), MassBank of North America (MoNA) library (MassBank of North America, 2022), and NIST ver. 2020 MSMS library (Mass Spectrometry Data Centre, 2022) [124,125].

### 3.8. Statistical Analysis

All metabolite datasets exported using Metaboscape ver. 2022b were examined using MetaboAnalyst 6.0 [126]. Prior to statistical evaluation, the data were log-transformed and auto-scaled to reduce heteroscedasticity and ensure comparability between variables of different magnitudes, as commonly recommended in metabolomics workflows [127]. Unsupervised multivariate analysis was performed using Principal Component Analysis (PCA) to assess overall clustering patterns and global differences between samples without prior class assumptions [128]. Enrichment analysis was conducted in MetaboAnalyst 6.0, utilising the Small Molecule Pathway Database (SMPD) to identify the major chemical classes of metabolites. To identify metabolites differentiating the two experimental groups, fold changes (FC) were calculated at each time point as the ratio of metabolite intensities in the fermented-preparation group relative to the non-fermented-preparation group. Metabolites with FC > 1.5 (higher in the fermented-preparation group) or FC < 0.67 (higher in the non-fermented-preparation group) and meeting the criteria of *p*-value < 0.05 (unpaired two-sample t-test) and false discovery rate (FDR) < 0.05 (Benjamini–Hochberg correction) were considered significantly altered. These thresholds were selected to balance sensitivity to biologically meaningful changes with control of false discoveries and are consistent with commonly applied statistical practices in exploratory untargeted metabolomics studies related to microbial community architecture and microbial ecosystem dynamics [129].

## 4. Conclusions

This study evaluated the impact of bioactive components derived from apple pomace fermented at low temperature on human gut microbiota. In a dietary model supplemented with apple pomace fermented by *K. barnettii* D1, a marked expansion of the mucin-degrading genus *Akkermansia* was detected, consistent with enhanced barrier-associated functionality. This intervention also promoted a more physiologically balanced short-chain fatty acid profile relative to the untreated control. Shotgun metagenomics further substantiated a more pronounced restructuring of community architecture and functional potential following bioprocessing, shifting the ecosystem toward a metabolically advantageous state.

Fermentation selectively enriched *Akkermansia*, *Coriobacteriaceae*, and *Parabacteroides*, while suppressing *Bifidobacterium*, *Klebsiella*, *Serratia*, and *Raoultella*. This is indicative of a transition toward a consortium preferentially engaged in mucin turnover and polyphenol biotransformation. Integrated metabolomic and SCFA profiling revealed diminished phenolic end products of proteolysis alongside progressive normalisation of acetate, propionate, and butyrate proportions, which more closely approximated reference ranges than those observed in controls.

Collectively, these taxonomic and functional reconfigurations suggest that pre-fermentation reprograms microbial metabolism from a predominantly fibre-driven, less specialised assemblage toward a mucin- and SCFA-oriented network aligned with improved epithelial barrier integrity and metabolic homeostasis. These findings highlight the potential of fermented apple pomace as a strategy for precision modulation of gut ecosystems, while highlighting the necessity for rigorous evaluation of both salutary and unintended ecological consequences in the development of functional fermented foods, in accordance with the principles of the circular economy.

## Data Availability

The raw reads were deposited in the NCBI database under the BioProject accession PRJNA1381616 and BioSample accessions SAMN54116487–SAMN54116492.

## Supplementary Materials

The following supporting information can be downloaded at https://www.mdpi.com/article/10.3390/ijms27072960/s1.

## Abbreviations

SCFA	Short-chain fatty acid
SHIME^®^	Simulator of the Human Intestinal Microbial Ecosystem^®^
MAGs	Metagenome-assembled genomes
SRGs	Species-level representative genomes
