# Supplementary figures and images for: Apple Pomace Fermented with Non-Saccharomyces Yeast as a Factor Modulating Gut Microbiota
Source: Int J Mol Sci. 2026 Mar 24;27(7):2960. doi: 10.3390/ijms27072960 (PMC13072911; doi:10.3390/ijms27072960)

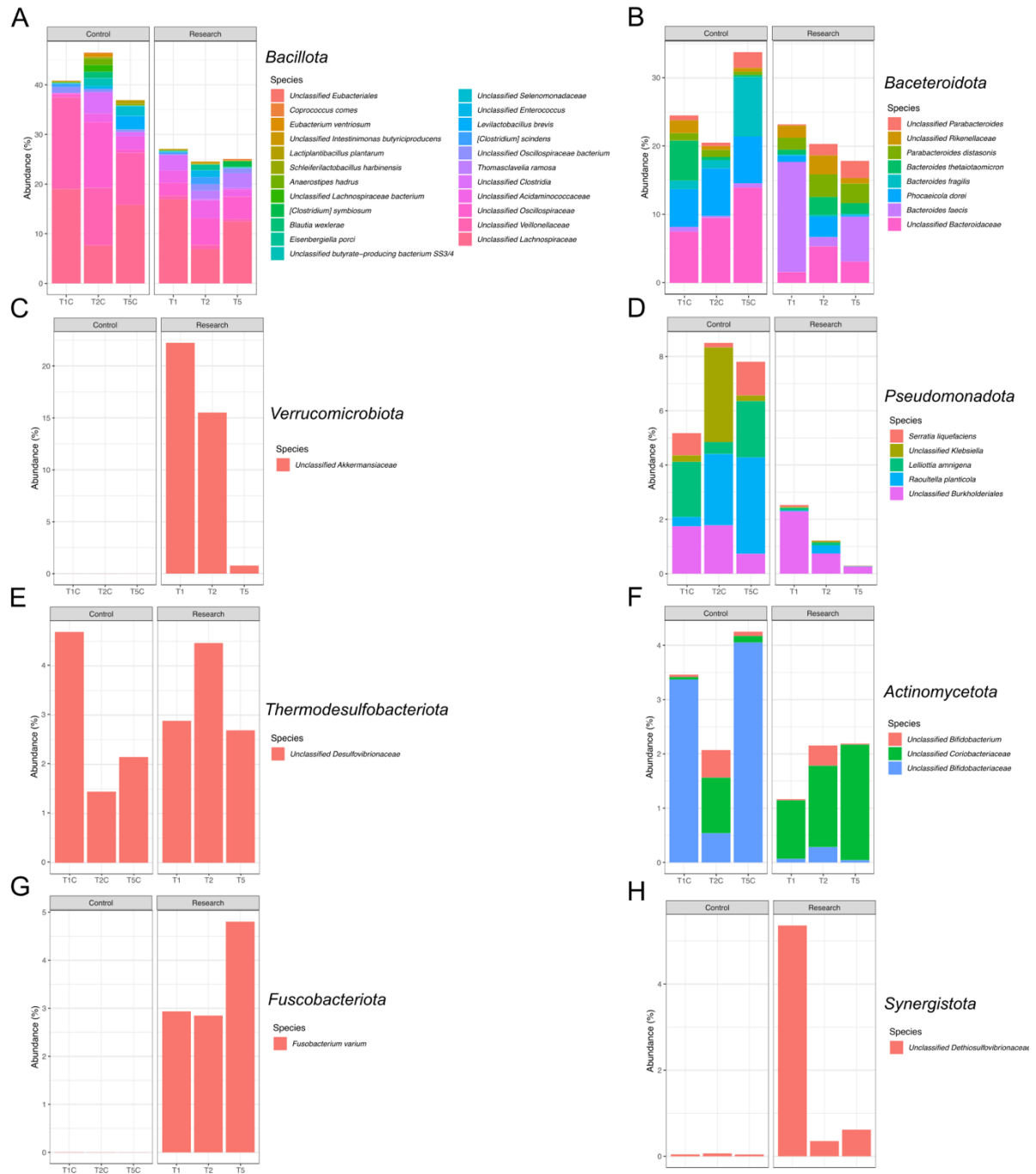

**Figure S1.** Relative abundance at species level for the phyla for strain-level genome bins (SGBs).

Supplement: Supplementary file 1 [file ijms-27-02960-s001.zip › 20260112_Supplementary Materials_SHIME.pdf]
